# Mr. Dyson, on Vaccine

**Published:** 1801-09

**Authors:** Theophilus Dyson

**Affiliations:** Surgeon, New Basinghall Street


### *To the Editors of the Medical and Phyfical Journal.*

Gentlemen,

In the relation of a cafe of Vaccine Inoculation by Mr. Pole, in page 340 of the Journal for April, he remarks, that in confequence of no progrefs being obfervable in the inflammation of the punctured part on the eighth day, he inoculated with the Variolous matter, and that in three days after, each incifion affumed the appearance of having taken effect. Both went on together until the *areola*, or inflammatory circle of the Vaccine furrounded that of the Variolous puncture, and thus influencing the conftitution, arrefted the further progrefs of Variolous infection on the fifth day. (An important medical fact.) The external marks of the latter now died away, the Vaccine only affecting the patient, and running its ordinary courfe in

 all all refpects, with its flight but ufual fymptomatic operation on the fyftem.

Mr. Pole then obferving of the fact of the inflammatory progrefs of the Vaccine Inoculation remaining almoft imperceptible or inactive for the firft eight days, that he did not remember an inftance to have occurred before, I extract for his information, as well as for medical practitioners in general, the following note from a memorandum-book I keep of patients inoculated with the Vaccine matter; viz. "November the 10th, 1800. Inoculated Mr. Barland's child, of Princes Street, Lothbury, ten months old; had no appearance of the Vaccine Inoculation having taken effect until between the eighth and tenth days; inflammation of the punctured part then came on, and ran the ufual courfe." The arm afterwards, I remember, exhibited a remarkable fine puftule: but when I called on the eighth day, fo little and hopelefs was the appearance of the infection having taken place, that I told the parents I fhould inoculate the child again on the day but one after, viz. on the tenth day. I paid my vifit for that purpofe, and was very agreeably furprifed to find the punctured part inflaming, and proceeding as above related.

I beg leave to take this opportunity to notice the like phænomenon in the Variolous inoculation, which others no doubt may have obferved. Both my partner, Mr. Price, and myfelf, have met with feveral inftances in the courfe of twenty years practice, of the Variolous matter remaining inactive on the incifed part for eight or ten days or longer, without fhewing any appearance or advancement of inflammation, more than might be excited by irritation on the fkin with any cutting inftrument. After fuch period the inflammatory rednefs and puftular prominence have began to take place, and gone on regularly in their natural courfe, the febrile and eruptive fymptoms of the Variola fucceeding in the uniform and ufual way.

*New Basinghall Street,*

*June* 23, 1801. THEOPHILUS DYSON, Surgeon,

